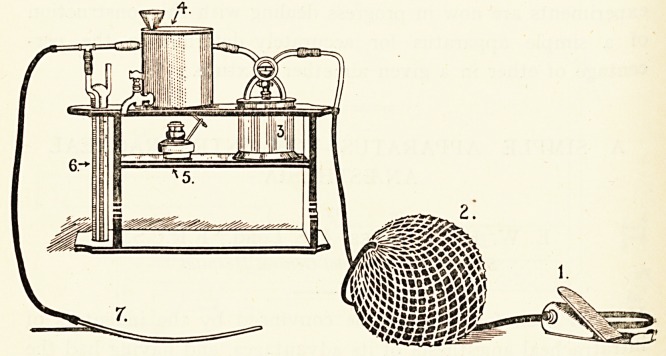# A Simple Apparatus for Intratracheal Anæsthesia

**Published:** 1913-12

**Authors:** E. W. Hey Groves

**Affiliations:** Surgeon to the Bristol General Hospital.


### A SIMPLE APPARATUS FOR INTRATRACHEAL ANÆSTHESIA.

BY

E. W. Hey Groves, M.S. Lond., F.R.C.S.,

*Surgeon to the Bristol General Hospital.*

---

Having been for a long time convinced by the inventors of intratracheal anæsthesia of its advantages, and having had the privilege of seeing Dr. Shipway use the method at Guy's Hospital, I determined to acquire a suitable apparatus. I was deterred from buying any apparatus, either American or English, which was upon the market, because of the bulk, complexity, and expense of all of these.

The Fig. shows the apparatus. (1) is a small foot pump filling distensible rubber bag (2) which keeps up a constant air stream. This passes into or over the ether bottle (3) by means of a four-way tap. This tap allows all the air to pass through the ether chamber, or to go on without any mixture with ether, or any intermediate condition of air and ether mixtures. From the ether chamber the air passes in a spiral tube contained in a hot-water tank (4), which can be kept heated by the spirit lamp (5) underneath. From the heating tank the air passes over a glass pressure gauge (6), which also acts as a safety valve. This is merely a water gauge, in which the glass tube connected with the air stream is covered by seven or eight inches of water. If the air pressure in the apparatus should rise above this, which corresponds to 14 to 16 mm. of mercury, the air escapes by bubbling out at the bottom end of the submerged tube. Lastly, a tube and catheter forming the delivery limb of the apparatus completes the equipment.

About the efficiency of the apparatus I have no doubt, except in the one and important point of the air pump. The one which I have used and figured is too small, and is liable to impose an undue strain on the anæsthetist during a prolonged operation.

A large foot bellows, with big rubber gas-bag and $\frac{3}{8}$-in. delivery tube, is required for an ample air supply without undue exertion, and, of course, for hospital work, some form of motor blower will always be used.

Discussion.—The three foregoing descriptions of apparatus were demonstrated at the meeting of the Bristol Medico-Chirurgical Society on November 12th.—The President said that it was a great advantage to have seen together the three different pieces of apparatus shown by Mr. Shipway, Mr. Stock and Mr. Groves, and he thanked Mr. Shipway on behalf of the Society for his interesting demonstration, stating that he had taken the opportunity of seeing Mr. Shipway's method in practice that afternoon at the Bristol General Hospital. That intratracheal anæsthesia was of great value in many cases he knew from his own experience of the method, since Mr. Stock had introduced it at the Royal Infirmary for several operations in his clinic earlier in the year. He had been particularly struck with the quiet breathing and absence of all respiratory embarrassment from blood getting into the trachea, in fact even with a pool of blood in the pharynx there was no need to mop, as far as respiration was concerned. But at the Royal Infirmary they had found no difficulty in introducing the tube into the trachea either by touch or by the direct method of inspection, and he thought that while the direct laryngeal tube was simple and preferable in many patients, in old patients with short thick necks, in whom complete extension of the head was unattainable, the direct laryngeal tube was likely to present some difficulty in practice.—Mr. E. W. HEY GROVES said it was the smooth type of anæsthesia produced by the method which first led him to advocate its application. That afternoon Mr. Shipway had employed the method at the Bristol General Hospital on a patient of the speaker's, who had chronic left empyema and paralysis of the left vocal cord. He thought that it was through the application of the method that the operation had been made possible. The speaker also showed an apparatus he had himself had constructed for the administration of ether in this way. He hoped the method would prove available for cleft palate operations.—Mr. C. A. MORTON expressed the gratitude felt to Mr. Shipway for his demonstration. He did not feel convinced that the method diminished shock to the degree alleged, but in cases of respiratory embarrassment, as in goitre, it was extremely useful.—Mr. STOCK inquired whether preliminary narcotisation by hypodermic injection was recommended by the demonstrator, and if he considered it better to have the eye of the catheter used as a lateral one rather than terminal.—The PRESIDENT asked why the catheter was passed down to the bifurcation of the trachea. He thought the indirect method of inserting the catheter in some cases would be preferable to the direct method. He asked if a nasal catheter would not be best in some cases.—Mr. SHIPWAY, in replying, said it was absolutely necessary to pass the catheter to the bifurcation so as to have the "dead space" above it. He advised a preliminary injection of morphine and atropine, and applied five per cent. cocain solution to the pharynx and upper aperture of the larynx. He preferred the direct method of passing the catheter, and rarely needed to extend the head, passing the laryngoscope from the side of the mouth. He believed tracheitis was guarded against by having a lateral eye to the catheter. In cleft palate he considered the patients were usually too young to have ether.

## Figures and Tables

**Figure f1:**